# Recommendations Regarding Tobacco Use and Secondhand Smoke Exposure from the Community Preventive Services Task Force

**Published:** 2013-09-20

Mass-reach health communication interventions target large audiences through television and radio broadcasts, print media, out-of-home placements (e.g., billboards, movie theaters, and point-of-sale), and digital media to change knowledge, beliefs, attitudes, and behaviors affecting tobacco use. The Community Preventive Services Task Force recommends mass-reach health communication interventions to reduce tobacco use and has posted new information about its systematic review at http://www.thecommunityguide.org/tobacco/massreach.html. The task force recommendation is based on strong evidence of effectiveness in 1) decreasing the prevalence of tobacco use, 2) increasing cessation and use of available services such as quitlines, and 3) decreasing initiation of tobacco use among young persons.

Established in 1996 by the U.S. Department of Health and Human Services, the task force is an independent, nonfederal, unpaid panel of public health and prevention experts whose members are appointed by the Director of CDC. The task force provides information for a wide range of decision makers on programs, services, and policies aimed at improving population health. Although CDC provides administrative, research, and technical support for the task force, the recommendations developed are those of the task force and do not undergo review or approval by CDC.

